# Microcystins Exposure and the Risk of Metabolic Syndrome: A Cross-Sectional Study in Central China

**DOI:** 10.3390/toxins16120542

**Published:** 2024-12-14

**Authors:** Shuidong Feng, Yuke Zeng, Fengmei Song, Minxue Shen, Fei Yang

**Affiliations:** 1 Department of Epidemiology and Health Statistics, The Key Laboratory of Typical Environmental Pollution and Health Hazards of Hunan Province, School of Public Health, Hengyang Medical School, University of South China, Hengyang 421001, China; shuidong_f@hotmail.com (S.F.); yukezeng09@gmail.com (Y.Z.); songfengmei2020@163.com (F.S.); 2 Hunan Provincial Key Laboratory of Clinical Epidemiology, Department of Social Medicine and Health Management, Xiangya School of Public Health, Central South University, Changsha 410000, China

**Keywords:** microcystins, metabolic syndrome, mediation, cross-sectional study

## Abstract

A growing body of evidence indicates that microcystins (MCs) exposure may cause metabolic diseases. However, studies exploring the effects of MCs exposure on the risk of metabolic syndrome (MetS) in humans are currently lacking, and the underlying mechanisms remain unclear. Here, we conducted a cross-sectional study in central China to explore the effect of serum MCs on MetS, and assessed the mediation effects of the inflammation biomarker, white blood cell (WBC) level, in this relationship. The relationships among MCs and WBC level and risk of MetS were assessed using binary logistic and linear regression. Mediation analysis was used to explore possible mechanisms underlying those associations by employing R software (version 4.3.1). Compared to the lowest quartile of MCs, the highest quartile had an increased risk of MetS (odds ratio [OR] = 2.10, 95% confidence interval [CI]: 1.19, 3.70), with a dose–response relationship (*p* for trend < 0.05). WBCs mediated 11.14% of the association between serum MCs and triglyceride (TG) levels, but did not mediate the association of MCs exposure with MetS. This study firstly reveals that MCs exposure is an independent risk factor for MetS in a dose–response manner, and suggests that WBC level could partially mediate the association of MCs exposure with TG levels.

## 1. Introduction

Metabolic syndrome (MetS) refers to a cluster of metabolic dysregulations, such as dyslipidemia, insulin resistance, elevated blood pressure, and abdominal obesity, which lead to a serious impact on the quality of life of patients. About one-quarter of the global population is affected by MetS, and the prevalence in China is expected to reach a third [1], with the prevalence of MetS continuing to increase [2,3,4,5]. Studies have demonstrated that MetS is strongly associated with hepatocellular carcinoma, cardiovascular disease (CVD), and other diseases [6,7,8,9,10], representing the greatest burden of non-communicable diseases worldwide [11]. The population attributable risks of MetS for the development of CVD and mortality are 17% and 7%, respectively [7,8]. MetS has emerged as a significant public health issue globally due to the enormous health burden it causes. Identifying controllable risk factors for MetS to prevent its related metabolic complications is essential.

Although risk factors for MetS include obesity, age, smoking, alcohol consumption, over-nutrition, poor economic conditions, and physical inactivity [2,4], its etiology remains to be elucidated. The acceleration of economic growth and industrialization has led to an increase in the number of environmental pollutants that threaten human health. Moreover, some of these pollutants have been identified as crucial contributors to the occurrence and development of MetS [12,13,14]. Nevertheless, the association of microcystins (MCs) exposure with MetS are relatively less studied. MCs, a type of waterborne toxin, are secondary metabolites of cyanobacterial blooms, which occur globally and pose a significant emerging public health threat [15,16]. It was demonstrated that MCs are present in water, soil, and vegetables [17]. Furthermore, they have the capacity to accumulate in aquatic wildlife and transfer to organisms at higher trophic levels [18,19,20]. MCs contamination in China is pervasive and acute, with eutrophication of about 80% of the inland lakes, and has recently garnered significant attention.

At present, at least 279 derivatives of MCs exist [21], inducing multi-organ toxicity in humans and wildlife [22,23,24]. Our previous epidemiological study has shown that exposure to MCs increases the risk of dyslipidemia [25]. Lin et al. reported that microcystin-LR (MC-LR) levels in early pregnancy are positively associated with gestational diabetes mellitus [26]. Guo et al. revealed that MC-LR can lead to increased blood pressure [27]. According to our previous study, MC-LR could lead to liver lipid accumulation [28]. However, the main focus in previous research has been on the individual components of MetS. Only one study investigated the relationship between MC-LR and MetS; nevertheless, it measured only MC-LR concentrations in external environments, which may not accurately reflect internal exposure levels [29]. Moreover, preliminary data have provided important evidence suggesting that MC-LR induced the production of reactive oxygen species (ROS), oxidative stress, and inflammatory reactions [30,31,32,33]. As an inflammatory marker, white blood cells (WBCs) secrete interleukins, interferons, tumor necrosis factors, and other cytokines, participating in the regulation of inflammatory response, and are widely reported to be associated with MetS [34,35,36,37]. However, the role of WBCs in linking MCs to MetS remains unknown.

Taken together, current evidence regarding the relationship between MCs and MetS remains limited and inconclusive, and the underlying mechanisms are unclear. It is required to conduct further studies to accumulate additional evidence, potentially facilitating policymakers to consider water pollution control as one of the strategies to control MetS. Therefore, a cross-sectional study was conducted in central China with the aim of investigating the association between MCs and MetS, as well as to assess the possible mediating role of WBCs, a marker of chronic inflammation, in this relationship.

## 2. Results

### 2.1. Characteristics of the Participants

Table 1 shows the demographic data of the 591 participants. A total of 199 (33.7%) met the criteria for MetS. Participants with MetS had a significantly higher average body mass index (BMI) than those without (*p* < 0.001). Moreover, there were significant differences in smoking status and gender between participants with and without MetS (*p* < 0.05). However, we did not find differences between the two groups with regard to age, education level, annual household income, alcohol consumption, or physical exercise.

### 2.2. MCs and WBC Levels

Table 2 presents the distribution of serum MCs concentrations and WBC levels. The median of the serum MCs and WBCs among participants were 0.15 µg/L and 5.98 × 10^9^/L, respectively. Participants with MetS exhibited significantly higher serum MCs levels (0.16 µg/L) compared to those without MetS (0.14 µg/L) (*p* = 0.003). Additionally, the WBC levels were significantly different between the two groups (*p* < 0.001). These results suggest that both serum MCs concentration and WBC levels may be associated with MetS.

### 2.3. Relationship Between MCs Exposure and MetS

**Figure 1:**
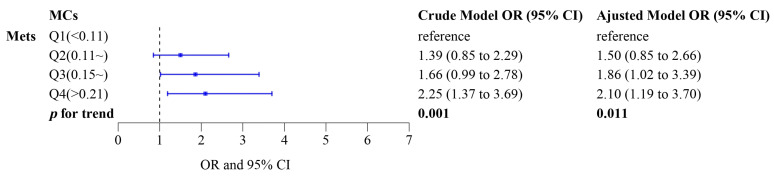
Odd ratios (ORs) and 95% confidence intervals (CIs) for metabolic syndrome (MetS) risk by quartiles of serum MCs concentration. Adjusted model: adjusting for gender, smoking status, and BMI.

**Figure 2:**
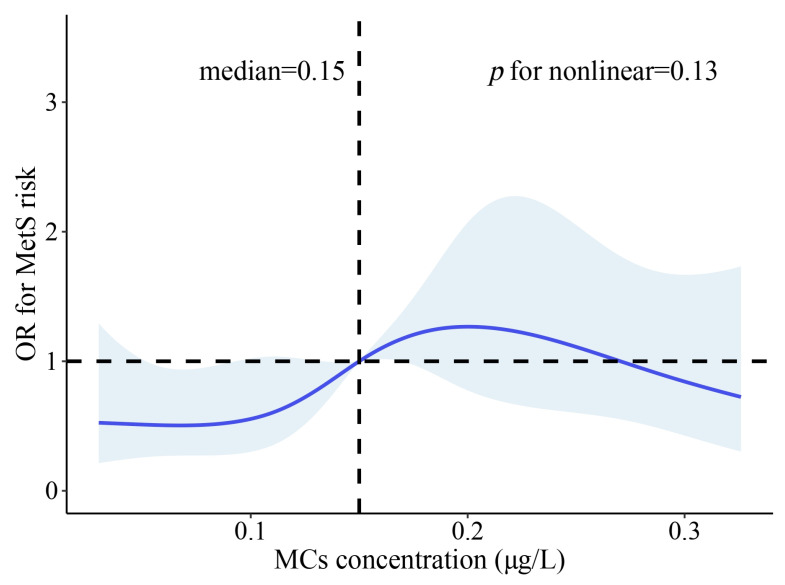
Restricted cubic spline (RCS) plot: relationship between serum MCs levels and MetS risk. The OR of MetS is represented by the solid blue line. Colored part represents the 95% CI. ORs were calculated based on continuous MCs values with adjusting for gender, smoking status, and BMI.

The association between exposure to MCs and MetS is shown in Figure 1. Compared to the lowest quartile of MCs, individuals who were exposed to the highest quartile had an increased risk of MetS (OR = 2.25, 95% CI: 1.37, 3.69). Similar results were observed in the adjusted model (OR = 2.10, 95% CI: 1.19, 3.70). MetS risk increased monotonically with increasing serum MCs quartiles (*p* for trend < 0.05) in both crude and adjusted models. Furthermore, restricted cubic spline (RCS) analysis showed no significant non-linear relationship between MCs and MetS (*p* for non-linear = 0.13) (Figure 2). Based on these results, a linear dose–response relationship was considered to exist between serum MCs concentration and MetS risk.

### 2.4. Mediation Analysis

The connection between MCs and WBC levels was investigated (Table S1). A doubling of serum MCs was associated with a 3.051% (95% CI: 0.120%, 6.067%; *p* < 0.05) increase in WBC levels. A significant positive association was found between each 1-unit increase in ln-transformed WBCs and MetS risk (OR = 3.696, 95% CI: 1.737, 7.864) (Table S2). The associations between WBC levels and individual MetS components (calculated as continuous variables) were further examined. Significant associations were observed between WBC levels and MetS components, with the estimated changes of 9.345 (95% CI: 6.050, 12.742) for diastolic blood pressure (DBP) and 36.162 (95% CI: 22.435, 51.428) for triglyceride (TG), respectively. Consistent results were found in adjusted models (Table S3).

**Figure 3:**
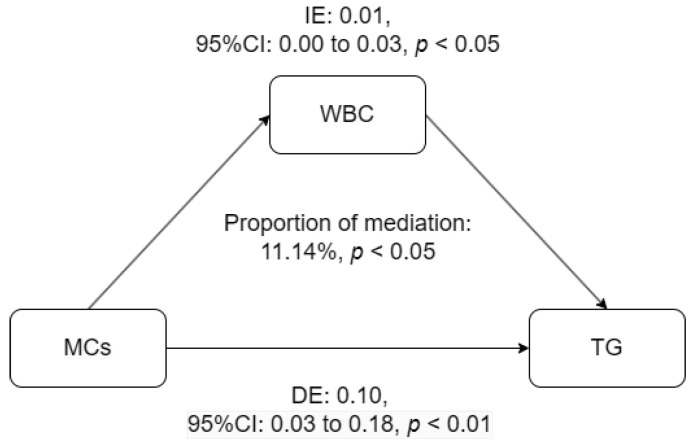
Mediation analysis. Abbreviations: IE, indirect effect; DE, direct effect; TE, total effect. A Non-parametric bootstrap mediation analysis was conducted on the association of serum MCs and TG levels with the mediators of WBC levels, adjusting for gender, smoking status, and BMI. In the mediation relationships, values on the center represent the mediation effect as a proportion of the TE, values on the top represent the IE (95% CI), and values on the bottom indicate the DE (95% CI).

The mediation analysis revealed that WBC levels could explain 11.14% of the connection between serum MCs and TG levels (Table 3). The indirect effect (IE) and the direct effect (DE) in the link between serum MCs and TG levels, mediated by WBCs, were 0.01 (95% CI: 0.00, 0.03; *p* < 0.05) and 0.10 (95% CI: 0.03, 0.18; *p* < 0.01), respectively (Figure 3). However, we did not observe that WBC levels mediated the relationship between MCs and MetS (Table 4).

## 3. Discussion

This study firstly evaluated the relationship between serum MCs concentration and risk of MetS. Our results suggest that exposure to MCs is an independent risk factor for MetS and exhibits a dose–response relationship. In addition, we observed that WBCs mediated 11.14% of the association of serum MCs exposure with TG levels. These results demonstrate the influence of environmental pollutants on MetS, and suggest that inflammation plays an important role in the association of MCs with TG levels.

To date, epidemiological evidence of an association of serum MCs concentration with MetS is lacking. As far as we know, only one previous study has been conducted to investigate the effects of MC-LR on MetS, and reported that MC-LR exposure (estimated daily intake) was not associated with MetS. However, their study estimated MC-LR exposure based on dietary intake, which might not accurately represent internal exposure levels [29], while our research directly measured serum MCs concentrations. Prior research had primarily examined the relationships between MC-LR and individual MetS components. Specifically, Lin et al. conducted a population-based study and found that serum MC-LR levels were associated with gestational diabetes mellitus. However, their study was limited to pregnant women in the first trimester and did not report the association of MC-LR with blood glucose levels [26]. In a previous study, we revealed that, compared to the lowest quartile of MCs, individuals exposed to the highest quartile had an increased risk of elevated TG and reduced HDL cholesterol (HDL-C) [25]. Chen et al. conducted a study on fishermen, revealing that 31.4% of those who drank water from MCs-contaminated lakes throughout their lives had abnormal TG indices. Nevertheless, their study did not investigate the association of MCs exposure with TG level [38]. An experimental study found that MC-LR significantly increased lipid deposition in the hepatocytes of obese mice [39]. Moreover, a cross-sectional assessment in a Pacific island community reported elevated levels of microcystin/nodularin in the urine of those with hypertension and hyperlipidemia [40]. Currently, although limited, the existing evidence generally suggests that a higher MCs level is linked with a higher OR of individual MetS components. Overall, our findings provided strong evidence supporting the association of MCs with the risk of MetS.

While the underlying mechanisms by which MCs influence MetS remain complex and not fully elucidated, insights from several studies have provided important clues. Zhang et al. reported that MC-LR exposure induced hepatic lipid metabolism disorder mediated by unsaturated fatty acid biosynthesis and peroxisome proliferator-activated receptor activation [41]. Additional research demonstrated that MC-LR exposure can induce apoptosis, autophagy, oxidative stress, and lipid accumulation in the liver [42,43]. Moreover, MC-LR exposure could cause hepatic endoplasmic reticulum stress through the alteration of mRNA and protein expression levels associated with endoplasmic reticulum stress signaling, as well as the factors and genes associated with hepatic lipid metabolism abnormalities in mice [44]. Our prior study had also shown that MC-LR exposure led to liver inflammation and disruption in hepatic lipid metabolism [28,39].

Higher WBC level was associated with an elevated risk of MetS and its individual components [37,45]. MC-LR exposure may cause oxidative stress, potentially followed by inflammation, increasing the number of WBCs [31,46,47], but evidence remains mixed [48]. Consequently, we hypothesized that MCs could induce MetS or individual MetS components by increasing WBC levels. Nevertheless, we found that the WBCs level mediated only 11.14% of the association for MCs and TG levels. In our study, the mediating effects of MetS and all other individual MetS components were not statistically significant, potentially due to low MCs concentrations. Our results indicate that only part of the link between MCs and TG level might be mediated by WBC levels, but elevated triglycerides is a critical component of MetS. Previous evidence also indicated that MC-LR can aggravate liver lipid metabolism disorders in obese mice by activating the PI3K/AKT/mTOR/SREBP1 signaling pathway in hepatocytes, resulting in elevated levels of key enzymes for lipid synthesis regulated by SREBP1-c, as well as blocking fatty acid β-oxidation [39]. Evidence exists that shows that MC-LR disrupts glucose, TG, and cholesterol metabolism, potentially linked to circulating thyroid hormone levels [49]. Additionally, it has been reported that MC-LR could down-regulate VEGFA and TGF-β expression via the AKT/m-TOR/HIF-1α pathway, which was linked to hypertension [27]. Meanwhile, as a powerful antioxidant, glutathione (GSH) could defend against oxidative stress [50]. It has been reported that GSH could also conjugate with MCs in liver tissue [51] and play a protective role against MC-induced liver injury [52].

This study was innovative in terms of exploring the relationship between serum MCs concentrations and MetS risk in adults, indicating a new environmental risk factor for MetS and enhancing our understanding of environmentally associated MetS risk factors. Furthermore, this study examined the mediating role of WBC levels in this relationship, thereby providing crucial insights for further research on underlying mechanisms.

Some limitations should be noted. First, cross-sectional designs inherently constrain the capacity to ascertain a causal relationship between variables. Nevertheless, the likelihood of reverse-causality (subjects with MetS choosing to reside in areas with high levels of MCs) is low, especially since the participants we recruited have been residents of this study area for over 5 years. Second, the collection of basic information through self-reporting may result in information bias. Third, despite our consideration of numerous confounding factors, the unmeasured confounding could persist. Fourth, the enzyme-linked immunosorbent assay (ELISA) could not detect MCs derivatives [53], which prevented us from finding out which derivatives might be correlated with MetS. Finally, while an elevated WBCs level is associated with inflammation, it could also be indicative of infection, trauma, or certain diseases [34]. Although WBCs level might not be as specific as genuine systemic indicators of inflammation, it correlates positively with these markers and is widely utilized in clinical practice [34,54,55]. Therefore, WBCs level serves as a viable marker of inflammation and could be used in research.

## 4. Conclusions

These findings suggest that MCs exposure is an independent risk factor for MetS in a dose–response manner, and although WBCs level does not mediate the association of MCs exposure with MetS, it could partially mediate the association of MCs exposure with TG levels. However, given the possible limitations of this study, a larger population sample size and prospective studies are necessary in order to obtain a more comprehensive understanding of the relationship between MCs concentrations and the MetS.

## 5. Materials and Methods

### 5.1. Study Design and Population

We conducted a cross-sectional, population-based study, building on prior research implemented in four regions in Hunan Province, China, from August 2016 to July 2017. Detailed information about the research has been described previously [56,57]. Participants were selected based on specific criteria and conditions. We recruited 730 residents (aged > 18) who had resided in the study area for more than 5 years. Comprehensive questionnaires were completed by participants, who also underwent blood draws and serum MCs tests. We ultimately enrolled 591 participants by excluding subjects with severe liver disease (n = 36), those lacking diagnostic data on MetS (n = 65), covariate information (n = 14), or data on blood biochemistry examination (n = 4), and those with MCs concentrations identified as outliers (defined as values above the upper quartile + 1.5 times the interquartile range [IQR], or below the lower quartile - 1.5 times the IQR) (n = 20).

The research protocol (Institutional Review Board number: 2018081028) was approved by the ethical review committee of Xiangya Hospital, Central South University. Prior to participating in the study, all subjects provided their written informed consent.

### 5.2. Data Collection

Trained investigators collected socio-economic data (e.g., age, gender, education level, and household annual income), lifestyle factors (e.g., smoking status, alcohol consumption, and physical exercise), and chronic medical history (e.g., diabetes, hypertension, and hyperlipidemia) through in-person interviews. Waist circumference (WC), systolic blood pressure (SBP), and DBP were measured by qualified medical personnel following standardized protocols. Furthermore, approximately 5 mL of blood, with and without an anticoagulant, was collected in tubes by qualified nurses from local hospitals. Dipotassium ethylenediamine tetraacetic acid (K2-EDTA, 2.0 mg/mL) anticoagulant tubes were used to collect blood samples when anticoagulants were used. The samples were subsequently centrifuged at 3000×*g* for 15 min using the centrifuge [Allegra 64R, Beckman Coulter, CA, USA], facilitating the separation of serum, blood cells, plasma, and blood clots. All samples were then collected into individual Eppendorf tubes and stored at a temperature of −80 °C.

### 5.3. Detection of Serum MCs

Serum MCs levels were measured using ELISA kits ((#20-0068), Beacon Analytical Systems Inc., Saco, ME, USA), adhering to the protocol of the manufacturer, which was described in a prior study [58]. The detection limit of MCs was 0.01 µg/L. MCs concentrations below their lower limits of detection (LODs) in the samples were imputed with a value equal to half of the detection limit [59]. Quality control of serum MCs detection was carried out by two copies of parallel and repeated determination. The recovery of serum samples ranged from 86.1% to 115.5%. The coefficient of variation ranged from 6.0% to 8.9%. The limit of quantification ranged from 0.1 µg/L to 2.0 µg/L.

### 5.4. Biochemical Measurements

WBCs level was determined in participants’ blood samples using the automatic hematology analyzer (LH750, Beckman Coulter, Brea, CA, USA). HDL-C, TG, and fasting blood glucose (FBG) level were assessed using the biochemical automatic detector (HITACHI 7600-020, Hitachi, Tokyo, Japan) following instructions provided by the manufacturer. WC, SBP, and DBP measurements were conducted by professionally trained investigators. WC was assessed using a tape measure positioned horizontally around the navel, while ensuring that the participant maintained smooth breathing and an upright posture. SBP and DBP were measured with the participant seated, after a minimum of five minutes of rest. Prior to measurement, participants were instructed to have an overnight fast.

### 5.5. Definition of the MetS

MetS was defined based on the criteria established by the International Diabetes Federation (IDF) [60]. In accordance with the diagnostic guidelines established by the IDF for MetS in China, an individual was typically defined as having MetS if he or she presented three or more of the following conditions: (1) a TG ≥ 150 mg/dL (1.7 mmol/L) or undergoing treatment for elevated triglycerides, (2) an HDL-C < 40 mg/dL (1.0 mmol/L) for men or < 50 mg/dL (1.3 mmol/L) for women or undergoing treatment for reduced HDL-C, (3) an FBG level ≥ 100 mg/dL (5.6 mmol/L) or undergoing treatment for elevated glucose, (4) a WC ≥ 90 cm for men or ≥ 80 cm for women, (5) an SBP ≥ 130 mmHg and/or DBP ≥ 85 mmHg or undergoing treatment for hypertension.

### 5.6. Covariates Assignment and Definition

In accordance with previous studies, the following variables were selected as covariates: age, gender (male, female), education level (primary or below, junior high school, high school, college or above), smoking status (no, yes), alcohol consumption (no, yes), household annual income (<10,000 CNY, 10,000 CNY–30,000 CNY, 30,000 CNY–50,000 CNY, >50,000 CNY), physical activity (no, yes), and BMI. A smoker was defined as an individual who had smoked at least 1 cigarette per day for a consecutive period of 6 months of his or her lifetime; all others were classified as non-smokers. An alcohol drinker was defined as an individual who had consumed alcohol at least once per week for 6 months of his or her lifetime; all others were classified as non-drinkers. The definition of regular physical activity was exercising at least twice a month for more than 20 min in the previous 6 months. BMI was calculated by dividing weight in kilograms by height in meters squared.

### 5.7. Statistical Analysis

The statistical analysis was conducted using the SPSS 26.0 software (SPSS, Chicago, IL, USA) and the R (version 4.3.1). Categorical variables were expressed in the form of frequencies (n) and percentages (%). Continuous variables were presented in the form of means ± standard deviations. For categorical variables, the chi-square test was used to assess differences between MetS patients and non-MetS patients; for continuous variables, the independent samples *t*-test or the Mann–Whitney U-test were selected according to the distribution. WBCs level, serum MCs concentration, and levels of TG, HDL-C, FBG, WC, SBP, and DBP were natural logarithm (ln)-transformed to achieve approximate normal distributions.

Binomial logistic regression models were used to estimate the OR and 95% CI of the MetS. MCs concentrations were divided into quartiles based on the study population. Furthermore, the linear dose–response relationship was assessed using trend tests by considering the median values of each quartile of MCs as a continuous variable. In addition, we used RCS with knots positioned at the 5th, 25th, 50th, 75th, and 95th percentiles of serum MCs distribution to explore the non-linear relationship between MCs and MetS risk. Models were adjusted for potential confounders based on known and suspected MetS risk factors, including gender, smoking status, and BMI.

Relationships among MCs, WBCs, and individual MetS components (calculated as continuous variables) were assessed using linear regression models. Regression coefficients (β) were transformed and represented the percent change in individual MetS components (calculated as continuous variables) per doubling of WBCs level using the following formula: [exp(ln2×β)−1]×100%. β were also transformed and represented the percent change in WBCs level per doubling of MCs concentration using the following formula: [exp(ln2×β)−1]×100%.

Mediation analyses were conducted to assess whether WBCs level mediated the association between serum MCs and MetS. The analysis utilized the R causal mediation analysis package [61]. A quasi-Bayesian Monte Carlo method with 10,000 simulations based on normal approximation was employed. The DE represented the effect of MCs exposure on MetS, independent of the mediator, and the IE represented the impact of MCs exposure on MetS mediated via WBCs level. The mediation proportion was calculated as the ratio of IE to the total effect (TE), and the suppression proportion was defined as the ratio of IE to the DE. An alpha level of 0.05 indicated statistical significance.

## Figures and Tables

**Table 1:** Demographical characteristics of the study participants.

Variable	N = 392 Non-MetS	N = 199 MetS	*p*
Age, years	53.7 ± 15.9	53.1 ± 14.2	0.807 ^a^
Gender, n (%)			0.005 ^b^
Female	109 (27.8%)	78 (39.2%)	
Male	283 (72.2%)	121 (60.8%)	
BMI, kg/m^2^	23.2 ± 3.2	27.0 ± 3.6	<0.001 ^a^
Education level, n (%)			0.597 ^b^
Primary or below	160 (40.8%)	78 (39.2%)	
Junior high school	87 (22.2%)	43 (21.6%)	
High school	96 (24.5%)	58 (29.1%)	
College or above	49 (12.5%)	20 (10.1%)	
Annual household income,			0.207 ^b^
CNY, n (%)			
<10,000	123 (31.4%)	56 (28.1%)	
10,000–30,000	130 (33.2%)	62 (31.2%)	
30,000–50,000	82 (20.9%)	57 (28.6%)	
>50,000	57 (14.5%)	24 (12.1%)	
Smoking, n (%)			0.005 ^b^
NO	190 (48.5%)	121 (60.8%)	
Yes	202 (51.5%)	78 (39.2%)	
Alcohol consumption, n (%)			0.272 ^b^
NO	298 (76.0%)	143 (71.9%)	
Yes	94 (24.0%)	56 (28.1%)	
Physical exercise, n (%)			0.973 ^b^
NO	224 (57.1%)	114 (57.3%)	
Yes	168 (42.9%)	85 (42.7%)	

^a^ From Mann–Whitney U test. ^b^ From chi-squared test. Abbreviations: MetS, metabolic syndrome; BMI, body mass index.

**Table 2:** The distribution of serum microcystins (MCs) and white blood cells (WBCs) in the participants.

			Percentile					
**Exposure**	**Group**	**Mean**	**5th**	**25th**	**50th**	**75th**	**95th**	***p*** ^**a**^
MCs (µg/L)	Total	0.16	0.04	0.11	0.15	0.21	0.29	0.003
	Non-MetS	0.15	0.04	0.10	0.14	0.19	0.29	
	MetS	0.17	0.05	0.12	0.16	0.22	0.30	
WBCs ($\times 10^{9}$/L)	Total	6.19	3.51	4.94	5.98	7.20	9.50	<0.001
	Non-MetS	5.96	3.40	4.72	5.72	6.97	9.12	
	MetS	6.65	3.79	5.40	6.45	7.74	10.30	

^a^ From Mann-Whitney U test.

**Table 3:** Mediating effects of WBC levels on the association between MCs and individual components of MetS.

Outcome	Total Effect (95% CI)	Direct Effect (95% CI)	Mediation Effect (%)	Suppression Effect (%)	*p*
WC	0.01 (0.00, 0.02) *	0.01 (0.00, 0.02) *	3.33	−	>0.05
SBP	−0.01 (−0.04, 0.01)	−0.02 (−0.04, 0.01)	−	5.37	>0.05
DBP	−0.00 (−0.03, 0.02)	−0.01 (−0.03, 0.02)	−	64.78	>0.05
FBG	0.05 (0.01, 0.08) *	0.05 (0.01, 0.08) *	−	2.01	>0.05
TG	0.11 (0.04, 0.20) **	0.10 (0.03, 0.18) **	11.14 *	−	<0.05
HDL-C	−0.04 (−0.07, −0.01) **	−0.04 (−0.07, −0.01) **	2.60	−	>0.05

* *p* < 0.05, ** *p* < 0.01. Abbreviations: WC, waist circumference; SBP, systolic blood pressure; DBP, diastolic blood pressure; FBG, fasting blood glucose; TG, triglyceride; HDL-C, HDL cholesterol.

**Table 4:** Mediating effects of WBC level on the association between MCs and MetS.

Outcome	Total Effect (95% CI)	Direct Effect (95% CI)	Mediation Effect (%)	Suppression Effect (%)	*p*
MetS	0.08 (0.01, 0.13) *	0.07(0.00, 0.12) *	13.35	-	>0.05

* *p* < 0.05.

## Data Availability

The data that support the findings of this study are available from the corresponding author upon reasonable request.

## Supplementary Materials

The following supporting information can be downloaded at: https://www.mdpi.com/article/10.3390/toxins16120542/s1, Table S1: Relationship between serum MCs concentration (ln-transformed) and WBC level (ln-transformed); Table S2: Relationship between WBC level (ln-transformed) and the odds ratio of MetS; Table S3: Relationship between WBC level (ln-transformed) and each components of MetS (ln-transformed).
