# Supplementary figures and images for: Microcystins Exposure and the Risk of Metabolic Syndrome: A Cross-Sectional Study in Central China
Source: Toxins (Basel). 2024 Dec 14;16(12):542. doi: 10.3390/toxins16120542 (PMC11679381; doi:10.3390/toxins16120542)

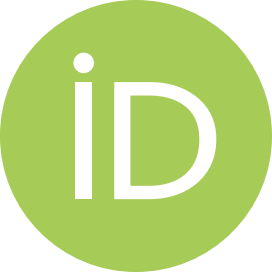

Supplement: Supplementary file 1 [file toxins-16-00542-s001.zip › Definitions/logo-orcid.pdf]
